# A degradable hydrogel scaffold incorporating the LL-37 for gingival soft tissue regeneration

**DOI:** 10.1093/rb/rbag131

**Published:** 2026-06-25

**Authors:** Jing Mao, Xiangyang Li, Kainan Liao, Longge Liu, Huimin Zheng, Yunong Cui, Zixuan Guo, Kexin Song, Junjie Zhao, Leping Wu, Weibo Zhang, Jialong Chen, Wei Li

**Affiliations:** College & Hospital of Stomatology, Anhui Medical University, Anhui Provincial Key Laboratory of Oral Diseases Research, Hefei 230032, China; College & Hospital of Stomatology, Anhui Medical University, Anhui Provincial Key Laboratory of Oral Diseases Research, Hefei 230032, China; Department of Biochemistry and Molecular Biology, Anhui Medical University (AHMU), Hefei 230032, China; College & Hospital of Stomatology, Anhui Medical University, Anhui Provincial Key Laboratory of Oral Diseases Research, Hefei 230032, China; College & Hospital of Stomatology, Anhui Medical University, Anhui Provincial Key Laboratory of Oral Diseases Research, Hefei 230032, China; College & Hospital of Stomatology, Anhui Medical University, Anhui Provincial Key Laboratory of Oral Diseases Research, Hefei 230032, China; College & Hospital of Stomatology, Anhui Medical University, Anhui Provincial Key Laboratory of Oral Diseases Research, Hefei 230032, China; College & Hospital of Stomatology, Anhui Medical University, Anhui Provincial Key Laboratory of Oral Diseases Research, Hefei 230032, China; College & Hospital of Stomatology, Anhui Medical University, Anhui Provincial Key Laboratory of Oral Diseases Research, Hefei 230032, China; College & Hospital of Stomatology, Anhui Medical University, Anhui Provincial Key Laboratory of Oral Diseases Research, Hefei 230032, China; College & Hospital of Stomatology, Anhui Medical University, Anhui Provincial Key Laboratory of Oral Diseases Research, Hefei 230032, China; College & Hospital of Stomatology, Anhui Medical University, Anhui Provincial Key Laboratory of Oral Diseases Research, Hefei 230032, China; College & Hospital of Stomatology, Anhui Medical University, Anhui Provincial Key Laboratory of Oral Diseases Research, Hefei 230032, China

**Keywords:** gingival recession, tissue regeneration, hydrogel scaffold, antibacterial, anti-inflammatory

## Abstract

Gingival recession exposes the roots, leads to dentin hypersensitivity, and heightens the progression of periodontitis, and poses a threat to oral health and appearance. Conventional treatment uses autogenous palatal grafts, requiring a second surgical site and adding patient discomfort. To overcome autograft limitations, this study developed a hydrogel scaffold as a potential biomaterial candidate for gingival soft tissue augmentation. We developed a multifunctional HA/SF hydrogel scaffold by incorporating EGCG and the antimicrobial peptide LL-37. It exhibits antibacterial, anti-inflammatory, antioxidant and pro-angiogenic activities. Its 3D network mimics the native extracellular matrix (ECM), enables minimally invasive delivery and conforms to irregular root surfaces. *In vitro*, it inhibits bacterial growth and adhesion, and significantly reduces pro-inflammatory cytokine expression in LPS-stimulated macrophages. Animal experiments in rats showed that the HA/SF/EGCG/LL37 scaffold significantly increased neovascular density, improved collagen fiber organization and enhanced anti-inflammatory and antioxidant effects versus controls. By balancing oral microbiota, dampening excessive inflammation and reducing oxidative stress, it represents a promising biologically active strategy that may facilitate gingival soft tissue regeneration.

## Graphical abstract

**Graphical Abstract:**
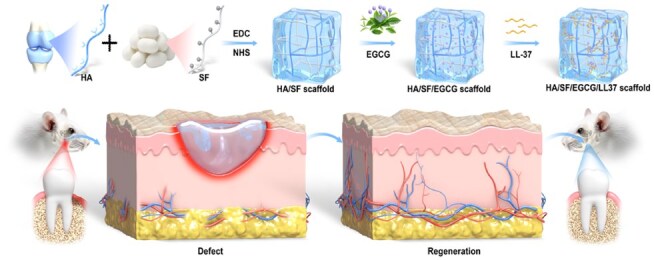

## Introduction

Gingival recession is a prevalent oral health condition that compromises esthetics and leads to dentin hypersensitivity, root caries and other complications [1], with epidemiological studies reporting prevalence rates of 81.1% for recession ≥1 mm, 48.4% for ≥3 mm and 16.2% for ≥5 mm [2]. Gingival recession is not caused by a single factor but results from the combined effects of multiple factors. Once it occurs, the original height cannot be restored through self-regeneration of the gingiva and alveolar bone [2, 3]. Although clinical management commonly relies on surgical techniques such as the coronally advanced flap (CAF) combined with a subepithelial connective tissue graft (SCTG) [4] or free gingival grafts [5], these approaches are constrained by donor-site morbidity, surgical trauma and patient discomfort, highlighting the need for minimally invasive regenerative strategies. Moreover, the moist and microbe-rich oral environment, together with foreign body reactions induced by implanted biomaterials, can lead to excessive reactive oxygen species (ROS) production, macrophage polarization toward the pro-inflammatory M1 phenotype and impaired angiogenesis, ultimately hindering tissue regeneration [6].Therefore, biomaterials capable of providing antibacterial, antioxidative, anti-inflammatory and pro-angiogenic functions are essential for effective gingival soft tissue regeneration. In light of the aforementioned limitations, various scaffold materials have been developed as alternative strategies for the regeneration of periodontal tissues damaged by inflammation [7–9]. An ideal material for periodontal regeneration should recruit regenerative cells and regulate their proliferation and differentiation, ultimately guiding the formation of functional periodontal tissues [10]. Accordingly, bioengineered scaffolds have been developed to mimic the structural and biochemical features of the extracellular matrix (ECM), thereby promoting tissue regeneration [11]. While synthetic hydrogels offer high tunability in material design, their potential biosafety concerns remain challenging. By comparison, natural biopolymer-based hydrogels possess superior biocompatibility and biodegradability, allowing for improved ECM mimicry and reduced adverse biological responses [12]. Furthermore, combinations of proteins/polysaccharides can partially simulate and replicate the structure and composition of the ECM [13]. Hyaluronic acid (HA) is a natural hydrophilic polysaccharide with excellent water retention and biocompatibility, but it suffers from poor mechanical properties and rapid degradation rates. Silk fibroin (SF) is a natural structural protein with outstanding mechanical strength and controllable degradation, widely used in biomaterials [14, 15]. However, it still has limitations such as material brittleness and insufficient space for cell growth [16]. Therefore, effectively combining HA and SF can enhance the properties of both. Studies have demonstrated that HA/SF composites can achieve significant results in tissue engineering fields beyond oral tissue regeneration, including bone [17], cartilage [18], skin tissue regeneration [19], spinal cord [20] and nerve [21] repair. Consequently, we hypothesize that HA/SF hydrogel scaffolds have the potential to serve as a tissue engineering material for oral soft tissue regeneration.

Antimicrobial peptides (AMPs) are small cationic molecules that play a vital role in the host’s innate immune response to infection, exhibiting broad-spectrum activity against pathogens [22, 23]. Their antibacterial mechanism primarily involves disrupting the structure of bacterial plasma membranes, causing massive leakage of cellular contents and ultimately leading to microbial death. Beyond direct bactericidal effects, AMPs are also less prone to inducing drug resistance and immune rejection [24]. LL-37 is currently the only human-derived antimicrobial peptide discovered. It can rapidly bind to lipopolysaccharides (LPS) on the surface of bacterial biofilms, neutralizing bacteria [25–28]. In addition to its bactericidal activity, LL-37 can also bind to Toll-like receptors (TLRs), inhibiting TLR signaling pathways and reducing the production of pro-inflammatory cytokines [29]. Moreover, studies have confirmed that LL-37 promotes wound healing by regulating cell proliferation and differentiation [30]. Its wound-healing activity is believed to stem from its ability to induce keratinocyte migration via transactivation of the epidermal growth factor receptor (EGFR) [31]. LL-37 is also involved in angiogenesis [32]; it binds to formyl peptide receptor-like 1 (FPRL1), subsequently activating downstream signaling pathways of FPRL1 to promote blood vessel formation [33], which is significant for gingival soft tissue regeneration [34–36].

However, the *in vivo* half-life of LL-37 is only about 60 min [37], making the achievement of sustained and controlled release of LL-37 at the surgical site of gingival recession a significant challenge. Therefore, therapeutic applications require a high loading capacity of LL-37. Epigallocatechin gallate (EGCG) is the most abundant polyphenolic compound in green tea, possessing potent anti-inflammatory and antioxidant physiological activities. It has been demonstrated to scavenge ROS while reducing the expression of pro-inflammatory factors and increasing the levels of anti-inflammatory cytokines in LPS-stimulated macrophages *in vitro* [38–40]. Consequently, EGCG can effectively reduce oxidative stress following periodontal surgery. Furthermore, there exists a natural and potent intermolecular interaction between EGCG and LL-37. LL-37 carries a positive charge under physiological conditions, while the phenolic hydroxyl groups of EGCG can partially dissociate in solution, conferring a negative charge [41]. This mutual attraction between opposite charges serves as the primary driving force for their binding, potentially further promoting the loading and release of LL-37 within the scaffold.

To address the limitations of autologous grafts and the complex challenges of the oral environment, this study developed a multifunctional HA/SF/EGCG/LL37 hydrogel scaffold that establishes a dynamically regulatable microenvironment for gingival soft tissue regeneration. The key novelty lies in leveraging natural electrostatic interactions between the positively charged basic amino acids of LL-37 and the negatively charged phenolic hydroxyl groups of EGCG. This synergistic co-assembly overcomes the critical translational bottleneck of LL-37's short *in vivo* half-life, enabling a sustained, controlled release system at the defect site. Rather than acting merely as a passive barrier, this biomimetic matrix imparts dual-action bioactivity: it actively neutralizes pathogenic threats through broad-spectrum antibacterial activity while simultaneously mitigating excessive ROS and dampening macrophage-mediated pro-inflammatory responses. By orchestrating the transition from a hostile inflammatory phase to an accelerated proliferative phase and promoting robust angiogenesis, this scaffold offers a compelling, clinically feasible strategy for gingival soft tissue wound healing and regeneration.

## Materials and methods

### Materials

Brain Heart Infusion (BHI) broth, Columbia blood agar plates, hemin, vitamin K and L-cysteine hydrochloride were all purchased from Qingdao Haibo Biotechnology. Silkworm cocoons, 2,2-diphenyl-1-picrylhydrazyl (DPPH), 2,2′-azino-bis(3-ethylbenzothiazoline-6-sulfonic acid) (ABTS), bovine serum albumin (BSA), Triton X-100 and lipopolysaccharide (LPS, L4391) were obtained from Sigma-Aldrich (Shanghai, China). Penicillin/streptomycin (P/S) and the bacterial live/dead staining dye (L13152) were provided by Thermo Fisher Scientific Inc. (Waltham, MA, USA). HA, 1-ethyl-3-(3-dimethylaminopropyl) carbodiimide hydrochloride (EDC), N-hydroxysuccinimide (NHS), Na_2_CO_3_, LiBr, CaCl_2_ and hydrogen peroxide (H_2_O_2_, 30 wt%) were all purchased from Aladdin (Shanghai, China). The RNA extraction kit was obtained from Escience Biotech. The Cell Counting Kit-8 (CCK-8), 2′,7′-dichlorodihydrofluorescein diacetate (DCFH-DA), Hoechst 33342 and the total antioxidant capacity assay kit (Ferric Reducing Antioxidant Power, FRAP method) were all purchased from Beyotime (Wuhan, China). Enzyme-linked immunosorbent assay (ELISA) kits were purchased from Elabscience.

### Cell culture

The murine fibroblast cell line (L929), human umbilical vein endothelial cells (HUVECs) and murine macrophage cell line (RAW 264.7) were obtained from the Cell Bank of the Chinese Academy of Sciences (Shanghai, China). L929 and RAW 264.7 cells were cultured in Dulbecco’s Modified Eagle Medium (DMEM, Gibco) supplemented with 10% fetal bovine serum (FBS, Gibco) and 1% penicillin/streptomycin. HUVECs were maintained in Endothelial Cell Medium (ECM, ScienCell). All cell lines were maintained at 37°C in a humidified atmosphere containing 5% CO_2_. Cells from passages 3–6 were used for all biological experiments described in this study.

### Preparation of the HA/SF/EGCG/LL37 hydrogel scaffold

HA powder was dissolved in deionized water with stirring to prepare a 3% HA solution. Silkworm cocoons were degummed by boiling three times in 0.5% Na_2_CO_3_ at 100°C for 30 min, rinsed thoroughly, then dissolved in CaCl_2_:CH_3_CH_2_OH:H_2_O. After dialysis (3 days) and filtration, a 3% SF solution was obtained. Based on previous structural evaluations of composite hydrogels [20], HA and SF solutions were mixed at a volume ratio of 6:4 (v/v). This specific ratio was selected to achieve an optimal balance between the superior water retention capacity of HA and the robust mechanical strength of SF without the need for additional optimization studies. EDC (40 wt%) and NHS (20 wt%) were added for crosslinking. The mixture was degassed, frozen at −20°C for 24 h and lyophilized for 48 h to yield the HA/SF scaffold.

### Characterization of the HA/SF/EGCG/LL37 hydrogel scaffold

#### Fourier transform infrared spectroscopy

Attenuated Total Reflectance Fourier Transform Infrared Spectroscopy (ATR-FTIR, Nicolet iS20, Thermo Fisher Scientific) was employed to analyse the functional groups of HA, SF, HA/SF scaffold, H/S/EGCG scaffold and H/S/E/LL37 scaffold. The scanning range for all samples was set from 4000 to 400 cm^−1^.

#### X-ray photoelectron spectroscopy analysis

Elemental analysis of the surfaces of the HA/SF scaffold, H/S/EGCG scaffold and H/S/E/LL37 scaffold was performed using an X-ray Photoelectron Spectrometer (XPS, ESCALAB 250Xi, Thermo Scientific). Initially, a wide survey scan from 0 to 1400 eV was conducted with a pass energy of 100 eV to record surface elemental distribution. Subsequently, high-resolution scans for individual elements were performed using a pass energy of 30 eV. The obtained spectra were processed and fitted using the Avantage software to determine elemental oxidation states. All spectra were calibrated based on the C 1s peak at 284.8 eV.

#### Scanning electron microscopy analysis

The morphological structure of the samples was examined using a Scanning Electron Microscope (SEM, Gemini 300, ZEISS). The HA/SF, H/S/EGCG and H/S/E/LL37 scaffolds prepared by the aforementioned methods were freeze-dried, sputter-coated with gold and observed under an acceleration voltage of 5 kV to image their internal microstructure. Pore size distribution was analyzed using ImageJ software.

To observe bacterial morphology, hydrogel scaffolds from each group were immersed in PBS and then co-incubated in BHI medium containing a bacterial suspension with a concentration of 10^6^ CFU/mL for 2 h. Subsequently, the bacterial suspensions were centrifuged, fixed, dehydrated, critical-point dried and examined via SEM.

#### Water absorption ratio

The dried hydrogel scaffolds were weighed (*M*_0_) and immersed in phosphate-buffered saline (PBS) at 37°C for 24 h. After carefully removing surface moisture with filter paper, they were weighed again (*M*_1_). The water absorption ratio (*W*) was calculated as:


$$W=\frac{M_{1}}{M_{0}}$$

#### Porosity

Porosity was determined via liquid displacement using anhydrous ethanol. The initial ethanol volume (*V*_1_) was recorded before immersing the scaffold. Following air removal under negative pressure, the total volume was measured as *V*_2_. After 20 min, the scaffold was extracted, and the remaining liquid volume (*V*_3_) was measured. Porosity (*P*) was calculated as:


$$P=\frac{V_{1}-V_{3}}{V_{2}-V_{3}}$$

#### Degradation weight remaining ratio

The dry weight of each hydrogel scaffold group was measured using an electronic balance and recorded as *W*_0_. Samples were placed in PBS and in PBS (pH 7.2) containing a 0.1 mg/mL proteinase K solution, respectively, and shaken in a constant temperature shaker at 37°C. The PBS and proteinase K-containing PBS solutions were refreshed every 24 h. Samples were retrieved at 1, 7 and 14 days, rinsed with deionized water, vacuum-dried and re-weighed. The weight measured at this stage was designated *W*_1_, and the degradation weight remaining ratio (DWR) was determined using the following formula:


$$\text{DWR}=\frac{W_{1}}{W_{0}}$$

#### Physicochemical of the hydrogel scaffolds

Composite hydrogel scaffolds with a uniform diameter of 5 mm and height of 5 mm were prepared. Compression tests were performed using a universal testing machine (AGS-X, Shimadzu Co, Japan) at a crosshead speed of 1 mm/min. The compressive modulus of each scaffold group was calculated based on the stress–strain curves.

#### *In vitro* release profile of LL-37 from the hydrogel scaffold

LL-37 release was measured by incubating the H/S/E/LL37 hydrogel scaffold in 1 mL PBS at 37°C with gentle agitation. At days 1, 4, 7, 10, 14, 21 and 28, the entire PBS volume was collected, replaced with fresh PBS, and stored at −20°C. Released LL-37 was quantified using an LL-37 ELISA kit, and cumulative release was plotted.

#### *In vitro* antibacterial properties evaluation

To evaluate the scaffold’s antibacterial properties, tests were conducted using *Staphylococcus aureus*, *Escherichia coli* and *Porphyromonas gingivalis*. The first two strains were cultured aerobically in BHI broth at 37°C for 24 h. *P. gingivalis* was grown under anaerobic conditions (80% N_2_, 10% H_2_, 10% CO_2_) at 37°C for 48 h in supplemented BHI broth (containing 5 μg/mL hemin and 10 μg/mL vitamin K). Bacteria were then harvested and standardized to a concentration of 1 × 10^6^ CFU/mL for subsequent experiments.

#### Bacterial survival assay

Hydrogel scaffolds from each group were immersed in PBS and then placed in BHI medium containing a bacterial suspension (10^6^ CFU/mL) for 2 h of co-incubation. After incubation, the bacteria were washed with saline, resuspended in fresh BHI medium and cultured overnight at 37°C. Finally, the optical density (OD) at 600 nm was measured using a microplate reader to assess bacterial survival rate, calculated as follows:


$$\text{Bacterial}\;\text{survival}\;\text{rate}\;(\%)=(\frac{OD_{\text{treatment}}}{OD_{\text{control}}})\times 100\%$$

#### Agar diffusion assay

The bacterial suspensions of S. aureus, E. coli, and P. gingivalis collected in the In Vitro Antibacterial Properties Evaluation section, upon reaching a confluency of over 80%, were serially diluted. Then, 100 μL of each diluted suspension was spread onto agar plates and cultured at 37°C for 24 h to observe colony formation. Additionally, scaffolds from each group were placed on the surface of agar plates uniformly spread with 10^8^ CFU/mL bacteria. After 24 h of incubation, inhibition zones formed around the scaffolds. The diameters of these zones were measured to evaluate the antibacterial properties of the hydrogels.

#### Live/dead bacterial staining

To visually investigate the effect of the hydrogel scaffolds on bacterial viability, scaffolds were immersed in PBS and then placed in BHI medium containing a bacterial suspension (10^6^ CFU/mL) for 2 h of co-incubation. The treated bacteria were stained using SYTO-9 and propidium iodide (PI), incubated in the dark for 30 min and then washed with saline to remove residual dye. Finally, the bacteria were resuspended in glycerol, placed on a glass slide and observed under a fluorescence microscope to capture images of bacterial morphology.

#### Biofilm formation inhibition assay

*S. aureus* and *E. coli* (10^6^ CFU/mL) were co-cultured with hydrogel scaffolds from each group in 6-well plates for 48 h to allow biofilm formation. The biofilms were then washed twice with PBS. The formed biofilms were stained with SYTO-9 dye and imaged under an optical microscope.

### *In vitro* biocompatibility assessment

#### Cell viability, proliferation and apoptosis assay

L929 cells were seeded uniformly in 48-well plates at a density of 2 × 10^4^ cells/well and co-cultured with the hydrogel scaffolds. After 1, 3 and 5 days, cell distribution on the scaffolds was assessed using Rhodamine 123 staining, and images were captured using a confocal laser scanning microscope (CLSM, Nikon A1, Japan). Cell proliferation was quantified using a CCK-8 kit at days 1, 3 and 5. After 5 days, cells were digested from the scaffolds for apoptosis analysis. Apoptotic cells were assessed by Annexin V/PI staining using a CytoFLEX flow cytometer (Beckman Coulter, USA).

#### Cell morphology

To further evaluate cell proliferation and adhesion morphology on the scaffolds, staining was performed using Rhodamine-phalloidin. L929 cells (1 × 10^4^ cells/mL) were co-cultured with hydrogel scaffolds for 3 days. Cells were fixed by adding 300 μL of 4% paraformaldehyde to each well. After washing with PBS, cells were permeabilized with 0.1% Triton X-100 for 15 min. Subsequently, the actin cytoskeleton of L929 cells was stained with 0.3% phalloidin for 60 min, and nuclei were stained with DAPI for 5 min. Images were captured using a CLSM.

#### Angiogenesis and wound healing assays

Fifty microliters of Matrigel were evenly spread in a 96-well plate and incubated for 1 h to allow solidification. HUVECs were then seeded onto the Matrigel at a density of 1 × 10^4^ cells/well. Cells were co-cultured with the hydrogel scaffolds using a transwell system. After 8 h of incubation, tube formation was observed and imaged using CLSM.

For the scratch assay, a confluent monolayer of HUVECs was established by seeding cells in 6-well plates at 3 × 10^5^ cells/well. Upon reaching a confluence of over 80%, the monolayer was scratched linearly with a sterile pipette tip. The resulting gap was imaged at 0, 12 and 24 h post-scratching under a CLSM, and the wound area was measured at these intervals using ImageJ for analysis.

### *In vitro* antioxidant capacity evaluation

#### DPPH radical scavenging activity

The free-radical scavenging rate was determined by employing the DPPH method. The hydrogel scaffolds were incubated with the DPPH working solution in the dark for 30 min, and then the absorbance of the mixture was measured at 517 nm. The formula below was used to calculate the DPPH free-radical scavenging activity:


$$\text{DPPH scavenging rate (\%)}=(1-\frac{A_{1}-A_{2}}{A_{0}})\times 100\%$$

*A*_0_, *A*_1_ and *A*_2_ denote the absorbances of the blank control, the sample and the sample–ethanol mixture, respectively.

#### ABTS cation radical scavenging activity

ABTS^+^ scavenging activity was measured. A pre-formed ABTS^+^ stock solution was prepared from 7.00 mM ABTS and 4.95 mM K_2_S_2_O_8_ (1:1, v/v) after 12 h in the dark. The stock was brought to an absorbance of 0.70 ± 0.02 at 734 nm by dilution with deionized water to create the working solution. In the test, 3 mL of this working solution was combined with 50 mg of hydrogel scaffold, kept in the dark for 30 min, and the absorbance was measured. The calculation of scavenging activity was performed using the equation below:


$$\text{ABTS scavenging rate (\%)}=(1-\frac{A_{1}-A_{2}}{A_{0}})\times 100\%$$

*A*_0_, *A*_1_ and *A*_2_ denote the absorbances of the blank control, the sample and the sample–deionized water mixture, respectively.

#### FRAP assay

The ferric reducing antioxidant power (FRAP) was determined. The FRAP stock solution and working solution were prepared in accordance with the instructions provided in the FRAP kit. The hydrogel scaffold sample (50 mg) was mixed with FRAP working solution (180 μL) and incubated at 37°C for 5 min. The absorbance of the reaction mixture was measured at 593 nm. FRAP values were expressed as FeSO_4_ concentration equivalents, calculated using a standard curve.

#### Intracellular ROS scavenging assay

Intracellular ROS levels were measured to evaluate the scaffolds’ antioxidant effect. L929 cells and HUVECs were co-cultured with scaffolds, then treated with 650 µM H_2_O_2_ for 24 h. After washing three times with PBS, cells were incubated with 10 µM DCFH-DA at 37°C in the dark for 20 min, followed by 5 min Hoechst staining in the dark for nuclear counterstaining prior to CLSM imaging.

#### Western blot analysis

The protein levels of Nrf2, HO-1 and NQO1 were determined via Western blotting. HUVECs, which were seeded at a density of 5 × 10^5^ cells/mL, were treated with the hydrogel scaffolds for 72 h. The cells were lysed in RIPA buffer containing protease inhibitors on ice. Proteins were separated using 10% SDS–PAGE, transferred onto PVDF membranes and then incubated overnight at 4°C with specific primary antibodies. Following a 1 h incubation with a secondary antibody at room temperature and subsequent washes with TBST, the bands were visualized using an ECL developing solution on a gel imaging system and analysed.

#### *In vitro* anti-inflammatory activity evaluation

RAW 264.7 cells were seeded in 24-well plates at a density of 5 × 10³ cells/mL. Five groups were established: control, LPS, HA/SF, H/S/EGCG and H/S/E/LL37. After cell attachment, all groups except the control group were treated overnight with 1 μg/mL LPS to induce an inflammatory response. Subsequently, each group was co-cultured with the corresponding materials for 24 h. The protein expression levels of interleukin-1β (IL-1β), tumor necrosis factor-α (TNF-α) and interleukin-6 (IL-6) in the cells were detected by Western blot analysis. Relative gene expression levels of IL-1β, TNF-α and IL-6 were measured by quantitative real-time PCR (qPCR) using Gapdh as an internal reference. The secretion of TNF-α and IL-6 was analysed using ELISA kits.

### Animal studies

#### Dorsal skin defect model

Animal experiments were approved by the Animal Ethics Committee of Anhui Medical University (Approval No. LLSC20231193). Healthy 8-week-old male Sprague–Dawley (SD) rats (weight: 220–280 g) were purchased from Liaoning Changsheng Biotechnology Co. Ltd. (Liaoning, China). Rats were randomly divided into three groups: blank control (PBS), H/S/EGCG and H/S/E/LL37. Rats were anesthetized with 3% sodium pentobarbital (1.8 mL/kg). Dorsal hair was shaved, and the skin was disinfected with iodophor. Three 10 mm full-thickness circular wounds were created using a round biopsy punch. The process of wound healing was documented on postoperative days 0, 3, 7 and 14. Digital images of the wounds were taken, and the wound-healing rate was calculated using ImageJ software.

#### Mucogingival surgery

SD rats were randomly divided into three groups: blank control (PBS), H/S/EGCG and H/S/E/LL37. After intraperitoneal injection of 1% sodium pentobarbital (0.1 mL/100 g), a longitudinal incision approximately 5 mm in length was carefully made in the palatal gingival mucosa near the midline, parallel to the gingival margin of the maxillary molars. The H/S/EGCG and H/S/E/LL37 scaffolds were customized according to the defect shape and placed at the gingival margin of the defect. One group served as a blank control without any material. The mandibular first molar sites, including alveolar bone and gingiva, were harvested at 21 days for analysis.

#### Histological evaluation of rat tissues

Following anesthetized euthanasia, tissue samples were collected from the dorsal wound site and the gingival region. The prepared paraffin sections were subjected to hematoxylin and eosin (H&E) staining and Masson’s trichrome staining. Additionally, immunofluorescence staining was performed to evaluate the expression and distribution of vascular endothelial growth factor (VEGF), type I collagen (COL‑I), TNF‑α and IL‑6.

### Statistical analysis

Experimental data were statistically analysed. All data are reported as mean ±SD. Differences between groups were assessed for significance using one-way analysis of variance (ANOVA), two-way ANOVA or Student’s *t*-test. Statistical significance is denoted as follows: *P < 0.05, **P < 0.01 and ***P < 0.001.

## Results and discussions

### Fabrication and characterizations of hydrogel scaffolds

**Figure 1:**
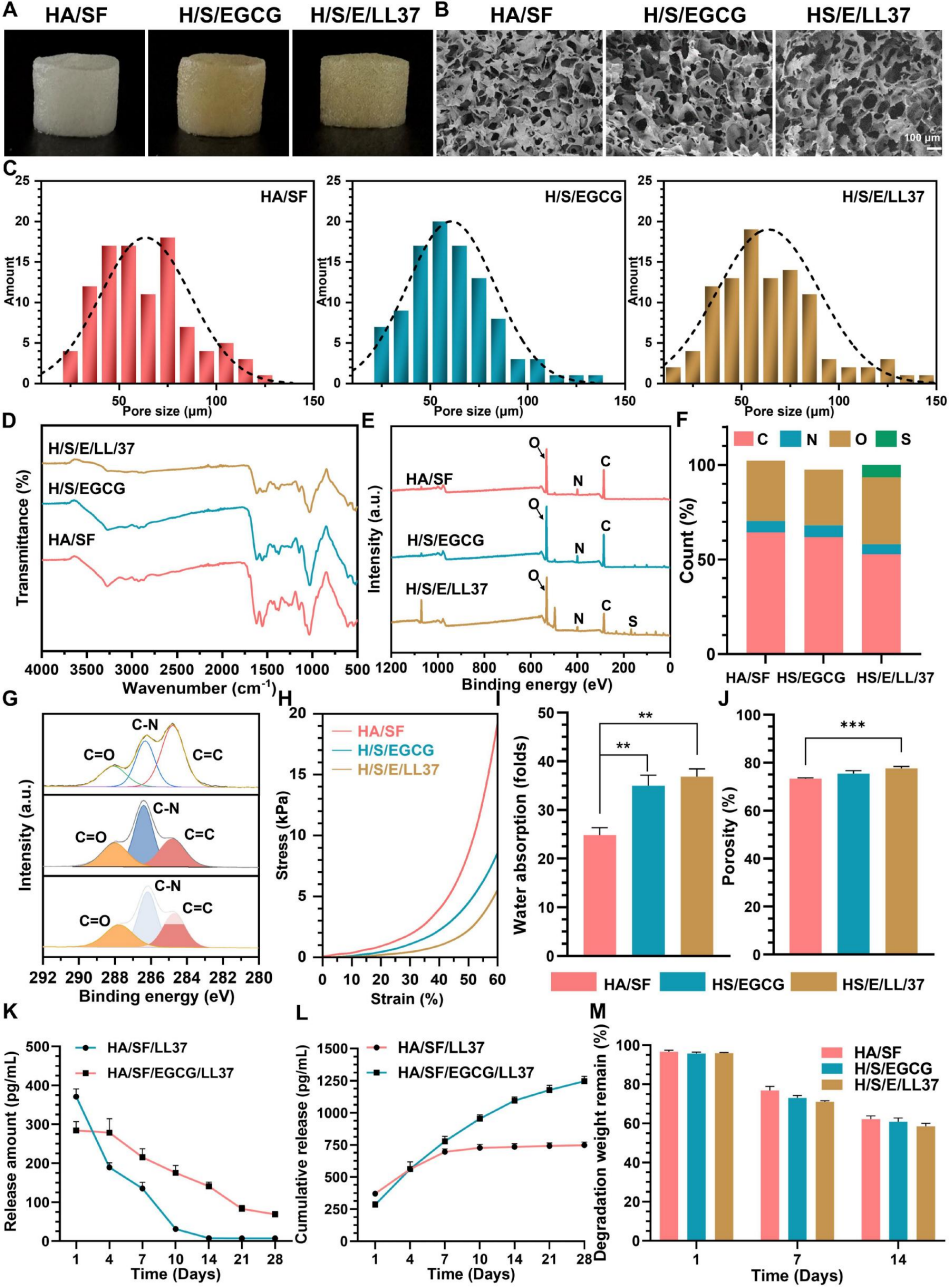
Fabrication and characterizations of hydrogel scaffolds. (**A**) Overview of HA/SF, HA/SF/EGCG and HA/SF/EGCG/LL37 hydrogel scaffolds. (**B and C**) SEM images and aperture analysis of scaffolds. Scale bars = 100 μm. (**D**) FTIR spectra of scaffolds. (**E–G**) XPS spectra of scaffolds (*n* = 3). (**H**) Stress–strain curve of scaffolds. (**I**) Water absorption rate (*n* = 3). (**J**) Porosity of hydrogel scaffolds (*n* = 3). (**K and L**) *In vitro* release of LL-37 in hydrogel scaffolds for 28 days (*n* = 3). (**M**) Degradation loss of hydrogel scaffolds in 0.05% trypsin solution at different time points (*n* = 3). *P < 0.05, **P < 0.01 and ***P < 0.001.

In recent years, hydrogel scaffolds have attracted considerable attention due to their ability to promote sustained release of substances and control their degradation [42]. In this study, we successfully prepared HA/SF hydrogel scaffolds via EDC/NHS-catalyzed amidation reaction, and used them as substrates to successfully load EGCG and LL-37. Figure 1A shows the general appearance of the HA/SF, H/S/EGCG and H/S/E/LL37 scaffolds, indicating that all groups possess similar external morphology. The microstructure and morphology of the hydrogel scaffolds were observed by SEM (Figure 1B). After freeze-drying, all scaffolds showed no obvious shrinkage and exhibited interconnected porous structures with pore sizes ranging from tens to hundreds of micrometers (Figure 1C). Compared to the other two groups (H/S/EGCG: 20–135 μm, H/S/E/LL37: 20–165 μm), the HA/SF hydrogel group exhibited the narrowest pore size distribution (between 20 and 120 μm), which may be attributed to the incorporation of EGCG and LL-37 affecting gel formation and structural organization. The rough scaffold surface and appropriate pore size are favorable for drug loading and controlled release, promoting nutrient exchange, vascularization, cell adhesion and proliferation.

To investigate the crosslinking of HA/SF and confirm successful loading of EGCG and LL-37, FTIR analysis was performed on the scaffold composition. As shown in Figure 1D, the peak at 1625 cm^−1^ results from the combined stretching vibration of the amide I band in SF and the C=O of the carboxyl groups in HA, indicating successful crosslinking between SF and HA to form a porous hydrogel scaffold. The –OH stretching vibration peak of HA/SF at 3284 cm^−1^ shifts to 3265 cm^−1^ (close to the EGCG stretching frequency of 3357.61 cm^−1^). Concurrently, in the synthesized H/S/EGCG scaffold, the peak intensity at this position weakens, the peak broadens and the distinction between the –OH at 3265 cm^−1^ and the N–H at 3056 cm^−1^ becomes less defined, confirming successful loading of EGCG onto the porous hydrogel scaffold. Furthermore, in the H/S/E/LL37 scaffold, the –OH peak shifts from 3265 cm^−1^ to 3261 cm^−1^, with decreased intensity and further broadening. The peaks at 3265 cm^−1^ and 3056 cm^−1^ nearly merge into a broader band, indicating stronger intermolecular interactions upon addition of LL-37 and successful loading of LL-37 onto the porous structure of the H/S/EGCG scaffold. X-ray photoelectron spectroscopy (XPS) was also employed to reveal elemental changes among different groups (Figure 1E and F). In the full range spectrum, the characteristic peak of S was observed to emerge following the introduction of LL-37. The deconvoluted C 1s spectra show three peaks at 284.8, 286.2 and 287.9 eV, assigned to C=C, C–N and C=O bonds, respectively. In H/S/EGCG, the C–N peak shifts to lower binding energy due to electron donation from EGCG’s –OH groups (Figure 1G). In H/S/E/LL37, the C=C peak intensifies markedly, reflecting incorporation of LL-37’s hydrophobic residues (e.g. leucine’s C–C/C=C) and peptide backbone contributions.

Due to frequent tissue movement and deformation, hydrogel scaffolds with weak mechanical properties cannot withstand external stresses imposed by the tissue environment. Thus, replicating the mechanical environment is a fundamental requirement in the design of soft tissue engineering grafts. The stress–strain curves of the hydrogel scaffolds demonstrate sufficient mechanical strength to resist gradually increasing deformation. At 60% compressive strain, the compressive strengths are 10.46, 8.67 and 5.60 kPa, respectively (Figure 1H). Furthermore, to explicitly assess the viscoelastic properties and mechanical stability of the scaffolds in a fully hydrated state, rheological testing was performed. As shown in Supplementary Figure S1, the storage modulus (*G*′) of the hydrogel scaffolds consistently exceeded the loss modulus (*G*″) across the tested frequency range. This demonstrates a typical solid-like elastic behavior, confirming that the crosslinked hydrogel network possesses robust mechanical stability and structural integrity in wet environments. In clinical applications, water absorption (swelling) capacity and porosity are important parameters of hydrogel scaffolds. Suitable water absorption and porosity facilitate the uptake of wound exudate, transport of nutrients and metabolites, and diffusion and release of drugs [43]. As shown in Figure 1I and J, all groups exhibit good swelling properties and high porosity, with swelling ratios ranging from 24.87 ± 1.49% to 36.85 ± 1.61%, and porosities from 73.40 ± 0.27% to 77.64 ± 0.78%.

Drugs can be incorporated into biodegradable scaffolds to achieve local delivery and induce tissue regeneration. The potential of using the H/S/E/LL37 scaffold as a local delivery platform was evaluated through drug release profiles. To determine whether the H/S/E/LL37 scaffold could control the release of LL-37, the release of LL-37 from different scaffolds was measured using an LL-37 ELISA kit. Figure 1K shows the *in vitro* release of LL-37 at each time point, and Figure 1L shows the cumulative release of LL-37 over 28 days. Among them, the H/S/LL37 hydrogel scaffold (only immersed in LL-37 solution) exhibited the highest release of LL-37 on day 1, tending toward complete release within 10 days. In the H/S/E/LL37 scaffold (immersed in solutions of both EGCG and LL-37), more cumulative release of LL-37 was detected, with a gradually slowing release curve that nearly plateaued around 28 days. This may be because LL-37 is rich in basic amino acids such as arginine and lysine, conferring an overall positive charge, which attracts the negatively charged EGCG through electrostatic interactions, facilitating their binding and the controlled release of LL-37. Compared to the immediate release of LL-37 from HA/SF/LL37, the controlled release of LL-37 from the H/S/E/LL37 hydrogel scaffold may better promote angiogenesis and tissue regeneration.

For tissue regeneration, biodegradability is essential-ideally, the material’s degradation rate should match tissue formation [44]. The degradation behavior of the hydrogel scaffolds was investigated in PBS and in PBS containing 0.1 mg/mL proteinase K solution (pH 7.2). As shown in Supplementary Figure S2, the hydrogel scaffolds remained highly stable in PBS, with no significant changes observed within 14 days, while all scaffolds exhibited appropriate biodegradability in the presence of proteinase K (Figure 1M). By day 14, the remaining weight percentages of the scaffolds ranged from 58.51 ± 1.56% to 62.18 ± 1.67%.

In addition, to accurately evaluate the structural retention of the scaffolds under simulated dynamic oral conditions, an *in vitro* dynamic stability test was conducted. The pre-weighed lyophilized scaffolds were immersed in artificial saliva (pH 6.8) and subjected to continuous mild agitation (60 rpm) at 37°C. As illustrated in Supplementary Figure S3, even under continuous shaking and repeated washing for up to 14 days, the H/S/E/LL37 hydrogel scaffold exhibited a steady and highly controlled degradation profile rather than a sudden catastrophic drop in mass. The remaining weight percentage confirmed that the composite network possesses robust washout resistance and excellent retention stability in a simulated dynamic intraoral environment.

### Antibacterial capacity of the HA/SF/EGCG/LL37 hydrogel scaffold

Following soft tissue defects and the implantation of gingival graft substitutes, bacterial infections can readily occur, leading to severe inflammation and hindering tissue healing. Furthermore, bacterial growth and colonization on the material can alter its intended performance. Given the complex microbial environment within the oral cavity, broad-spectrum antibacterial capability assays are essential to ensure the material’s effectiveness against various pathogenic bacteria and enhance clinical therapeutic outcomes. Therefore, *S. aureus* and *E. coli* were used to evaluate the scaffold’s antibacterial effects against Gram-positive and Gram-negative bacteria, respectively, to assess its broad-spectrum antibacterial activity. Additionally, *P. gingivalis*, a primary oral periodontal pathogen, was selected to further evaluate the scaffold’s antibacterial capacity.

**Figure 2:**
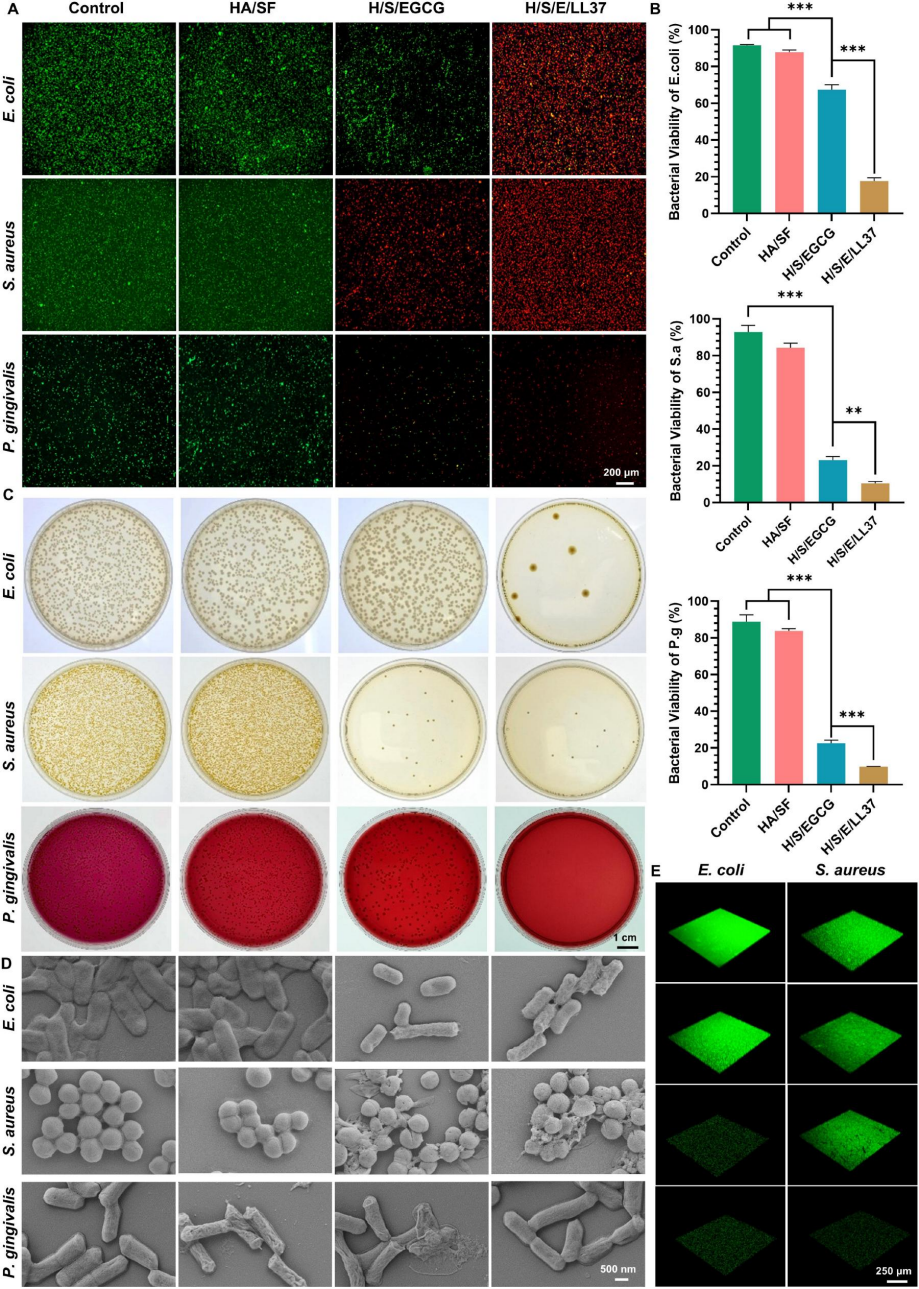
Antibacterial effects of hydrogel scaffolds. (**A**) Immunofluorescence staining of live/dead bacteria using SYTO 9 (green, live bacteria) and PI (red, dead bacteria). Scale bars = 200 μm. (**B**) Bacterial viability after co-culture with hydrogel scaffold (*n* = 3). (**C**) Liquid antibacterial bacterial plate culture results. Scale bars = 1 cm (*n* = 3). (**D**) Representative electron micrographs of bacteria seeded on the surface of H/S, H/S/EGCG and H/S/E/LL37. Scale bars = 500 nm. (**E**) Bacterial biofilm staining. Scale bars = 250 nm. *P < 0.05, **P < 0.01, ***P < 0.001.

As shown in Figure 2A, live/dead bacterial co-staining with SYTO 9 (staining live bacteria green) and PI (staining dead bacteria red) confirmed the superior antibacterial efficacy of the H/S/E/LL37 scaffold. Compared to other groups, H/S/E/LL37 showed a greater number of dead cells across all three bacterial strains. A previous study reported that LL-37 possesses broad-spectrum antibacterial properties, being effective against both Gram-positive and Gram-negative bacteria [45]. LL-37 has an amphipathic structure with hydrophilic and hydrophobic domains, allowing it to readily accumulate on bacterial surfaces. Its N-terminal region can interact with amphipathic phospholipid molecules, disrupting their arrangement, compromising the integrity of the cell membrane structure and function, thereby exerting bacteriostatic or bactericidal effects [46]. Consequently, the antibacterial activity of the scaffold was significantly enhanced after grafting with LL-37. Overall, the EGCG and LL-37 modified hydrogel scaffold exhibits desirable antibacterial activity, which is advantageous for its application in oral tissue repair within the complex microbial environment. The above findings were supported by statistical analysis of colony counts (Figure 2B), which yielded consistent conclusions.

As shown in Figure 2C, the control and HA/SF groups exhibited a large number of bacterial colonies, whereas the experimental groups loaded with EGCG and LL-37 showed a significant reduction in bacterial colony counts. This reduction was particularly pronounced in the group further loaded with LL-37, where only a few sporadic colonies were observed. It is noteworthy that the H/S/EGCG group demonstrated significantly weaker antibacterial activity against *E. coli* compared to its activity against *S. aureus* and *P. gingivalis*. This may be attributed to the denser, more complete outer membrane of *E. coli*, which is rich in well-organized LPS and exhibits lower permeability, forming an effective physical barrier that impedes the entry of EGCG. The ability of the samples to inhibit bacteria in the surrounding environment was also evaluated using the zone of inhibition test, as shown in Supplementary Figure S4. The width of the inhibition zone for the sample groups containing EGCG and LL-37 was significantly increased, aligning with the aforementioned research results.

The ultrastructural morphology of the bacteria was further characterized by SEM (Figure 2D). In the control and HA/SF groups, SEM images revealed typical morphological features: *E. coli* appeared as uniform short rods with intact cellular structure and no obvious lysis; *S. aureus* displayed intact spherical shapes, characteristically clustered together; *P. gingivalis* presented as short rods or coccobacilli with blunt ends, exhibiting intact and smooth cell membranes. In contrast, bacteria exposed to the H/S/EGCG and H/S/E/LL37 groups showed permanent damage to their membranes, characterized by rupture, distortion, wrinkling, shrinkage and in some instances, complete dissolution. These observations indicate that LL-37 impedes bacterial survival by compromising membrane integrity, leading to leakage of cytoplasmic contents. It is worth noting that EGCG also contributes to antibacterial activity through mechanisms such as disrupting membrane potential and inhibiting enzyme activity.

Unlike planktonic bacteria, biofilms provide enhanced protection for bacteria through extracellular polymeric substances (EPS), making them more prone to exhibiting greater antibiotic resistance. Therefore, the ability of the H/S/E/LL37 scaffold to eradicate biofilms formed by *E. coli* and *S. aureus* was further analysed using 3D fluorescence staining. As shown in Figure 2E, a significant reduction in green fluorescence highlights the capacity of H/S/E/LL37 to disrupt the biofilm structures of both *E. coli* and *S. aureus*. While our current results demonstrate robust antibacterial efficacy against single-species biofilms, the authentic oral environment is characterized by complex microbiomes. Therefore, future investigations utilizing dynamic multispecies oral biofilm models and prolonged time-kill evaluations will be necessary to fully validate the long-term clinical translational potential of this scaffold.

### Cytocompatibility of the HA/SF/EGCG/LL37 hydrogel scaffold

For biomaterials designed for long-term contact with internal tissues, cytocompatibility is of utmost importance for guaranteeing *in vivo* safety and efficacy. It serves as a crucial determinant that supports the material’s long-term stability and functionality within the host. Fibroblasts and endothelial cells play crucial roles in the gingival regeneration process. Therefore, L929 cells were seeded onto the hydrogel scaffolds to observe whether the material adversely affects cell growth and proliferation.

**Figure 3:**
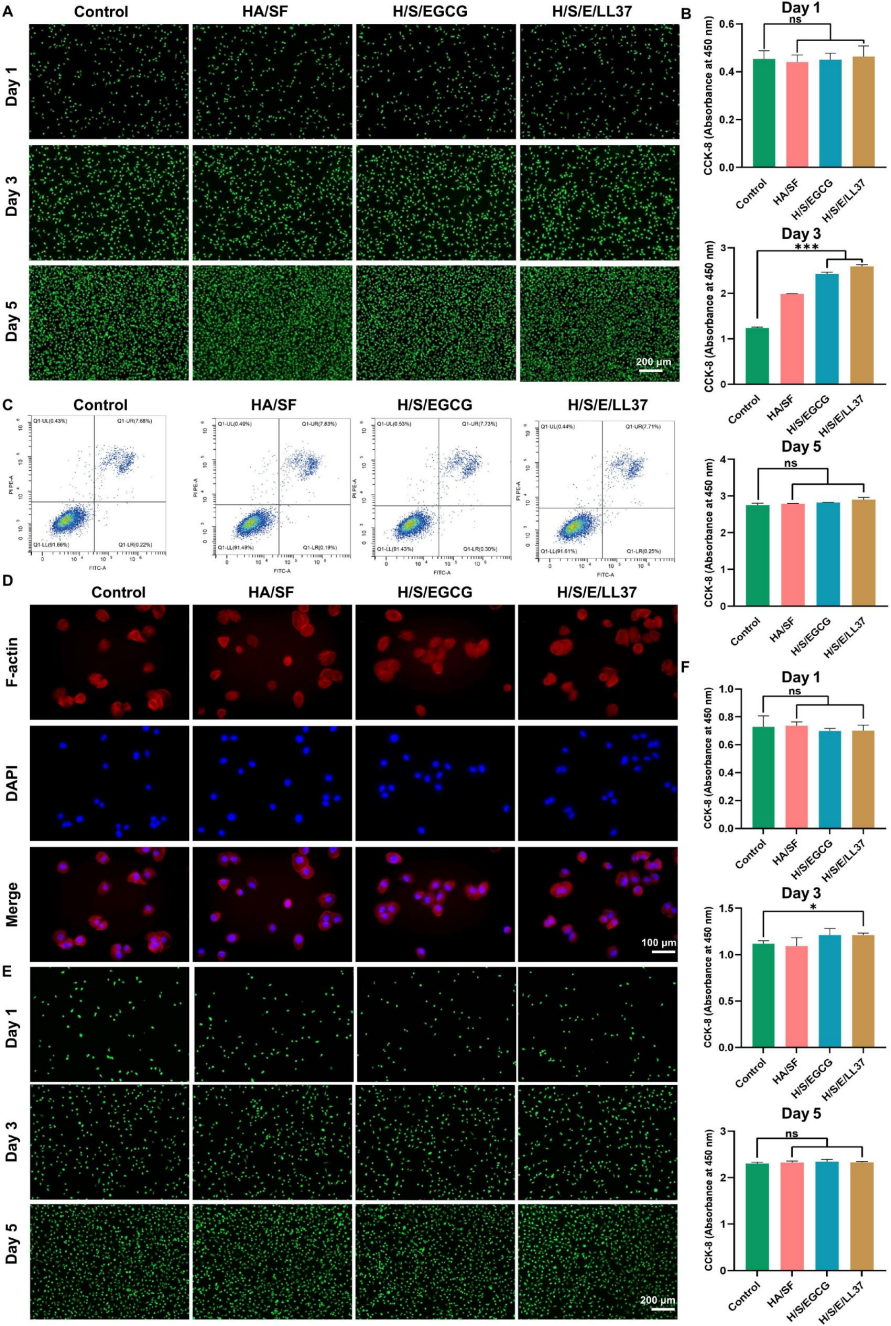
Biocompatibility of different hydrogel scaffolds. (**A**) Rhodamine 123 staining of L929 cells co-cultured with hydrogel scaffold for 1, 3 and 5 days. Scale bars = 200 μm. (**B**) CCK-8 assay results of L929 cells co-cultured with hydrogel scaffold for 1, 3 and 5 days (*n* = 3). (**C**) Cell death rate of L929 cells after 24-h co-culture with hydrogel materials, detected by apoptosis flow cytometry. The quantitative statistical analysis of the apoptosis rate is presented in Supplementary Figure S5 (*n* = 3). (**D**) Fluorescence staining of HUVECs co-cultured with hydrogel scaffold: cytoskeleton stained with F-actin (red) and nuclei stained with DAPI (blue). Scale bars = 100 μm. (**E**) Rhodamine 123 staining of HUVEC cells co-cultured with hydrogel scaffold for 1, 2 and 3 days. Scale bars = 200 μm. (F) CCK-8 assay results of HUVECs co-cultured with hydrogel scaffold for 1, 2 and 3 days (*n* = 3). *P < 0.05, **P < 0.01, ***P < 0.001.

First, Rhodamine 123 was used to observe the distribution of L929 cells within the scaffolds on days 1, 3 and 5. As shown in Figure 3A, the number of cells increased significantly in all the scaffold groups over the culture period. Rhodamine 123 staining was also performed on HUVECs seeded on the hydrogel scaffolds, and the results were consistent with those of L929 cells (Figure 3E). Subsequently, the CCK-8 assay was employed to evaluate cell proliferation on the scaffolds. Figure 3B shows that the OD values of L929 cells cultured on the composite hydrogel scaffolds exhibited an increasing trend on days 1, 3 and 5. Similarly, the OD values of HUVECs in Figure 3F also demonstrated a gradual upward trend. These results indicate that the composite hydrogel scaffolds have no adverse effects on cell proliferation.

Apoptosis of L929 cells within the three scaffold groups at the 5-day time point was further evaluated by flow cytometry. As shown in Figure 3C and Supplementary Figure S5, the total percentage of apoptotic cells showed no significant difference among the HA/SF group (7.83%), the H/S/EGCG group (7.73%), the H/S/E/LL37 group (7.71%) and the control group (7.68%). This indicates that the presence of EGCG and LL-37 did not significantly impact the apoptotic behavior of L929 cells, suggesting that the LL-37-loaded hydrogel scaffold is safe for potential clinical use.

To investigate whether the composite hydrogel scaffolds promote cell spreading, F-actin staining was performed to observe the actin distribution in HUVECs. After 1 day of culture, HUVECs exhibited similar spreading behavior across the HA/SF, H/S/EGCG and H/S/E/LL37 groups (Figure 3D).

### HA/SF/EGCG/LL37 hydrogel scaffold supports angiogenesis, cell proliferation and migration in vitro

**Figure 4:**
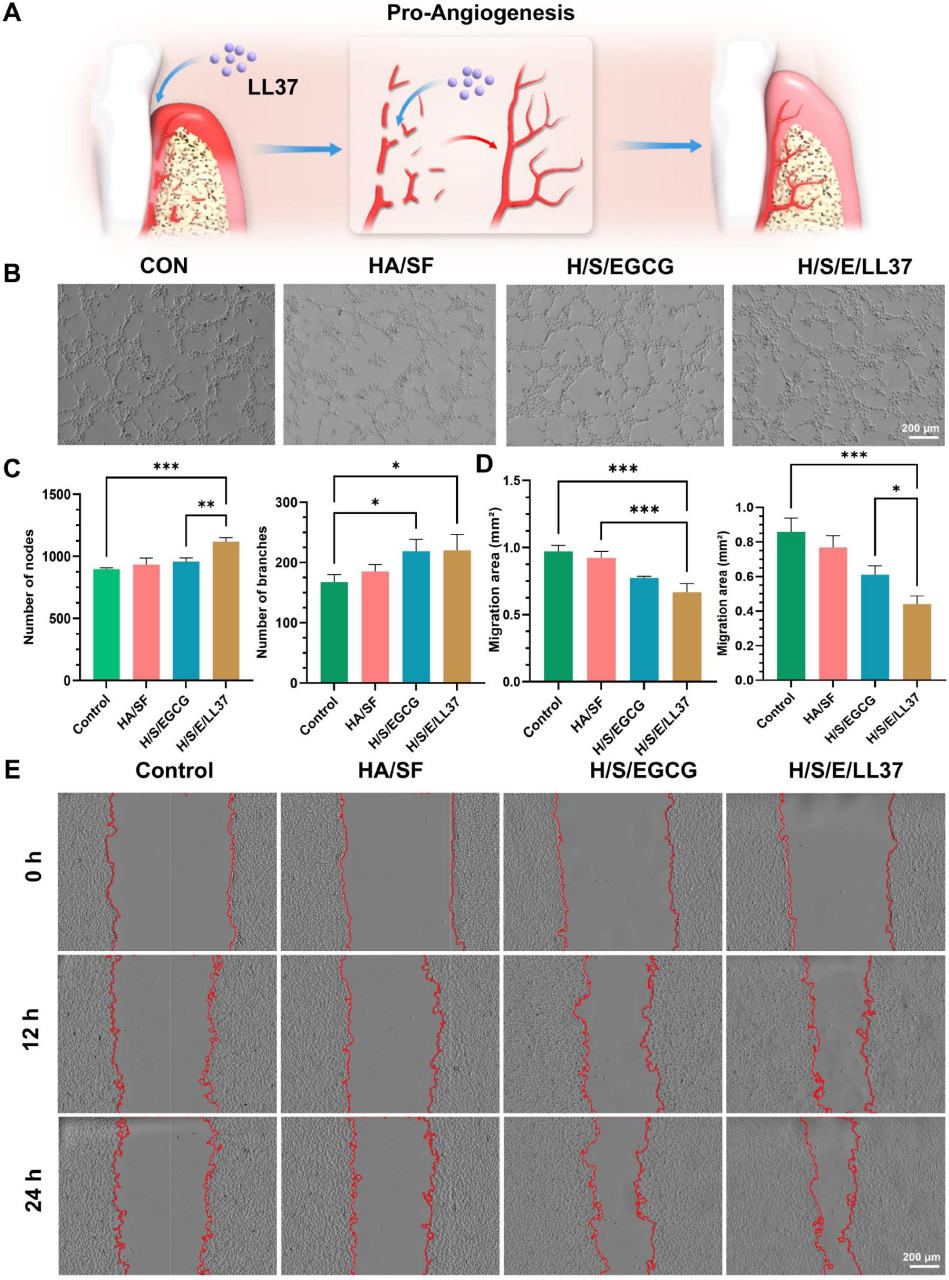
Effects of different hydrogel scaffold on angiogenesis and cell migration. (**A**) Schematic diagram illustrating the pro-angiogenic function of LL-37. (**B**) Digital images of *in vitro* tube formation assay after co-culture of HUVECs with hydrogel scaffold for 8 h. Scale bars = 200 μm. (**C**) Quantitative evaluation of formed tubular structures and junctions in the tube formation assay (*n* = 3). (**D and E**) Representative images of L929 cell migration at 12 and 48 h after incubation with hydrogel extracts, with corresponding statistical analysis of the scratch area. Scale bars = 200 μm (*n* = 3). *P < 0.05, **P < 0.01, ***P < 0.001.

Studies have shown that LL-37 can bind to specific receptors on vascular endothelial cells, such as formyl peptide receptor 2 (FPR2) and epidermal growth factor receptor (EGFR). This binding activates the MAPK/ERK and PI3K/Akt signaling pathways, thereby effectively exerting pro-angiogenic effects [47–49] (Figure 4A). To verify whether the H/S/E/LL37 hydrogel scaffold promotes angiogenesis in HUVECs and migration in L929 cells, a tube formation assay and a wound-healing scratch assay were performed using the transwell method. As shown in Figure 4B and C, the scaffold loaded with LL-37 promoted the formation of more tube-like structures and a greater number of junctions compared to the other groups.

As shown in Figure 4E, a noticeable migration of cells into the scratched area was observed in the H/S/E/LL37 hydrogel scaffold group compared to the other groups. Quantitative results (Figure 4D) indicated that wound healing was enhanced in the HA/SF, H/S/EGCG and H/S/E/LL37 groups compared to the control group, with the LL-37-containing scaffold group showing the highest degree of healing. Overall, these *in vitro* results demonstrate that the H/S/E/LL37 hydrogel scaffold can create a favorable environment for the adhesion, proliferation and migration of L929 cells.

These pro-angiogenic and pro-migratory effects are consistent with the known mechanism by which LL-37 binds FPR2 and activates downstream MAPK/ERK and PI3K/Akt signaling to promote angiogenesis [35, 47–49].

### Anti-inflammatory activity of the HA/SF/EGCG/LL37 hydrogel scaffolds

**Figure 5:**
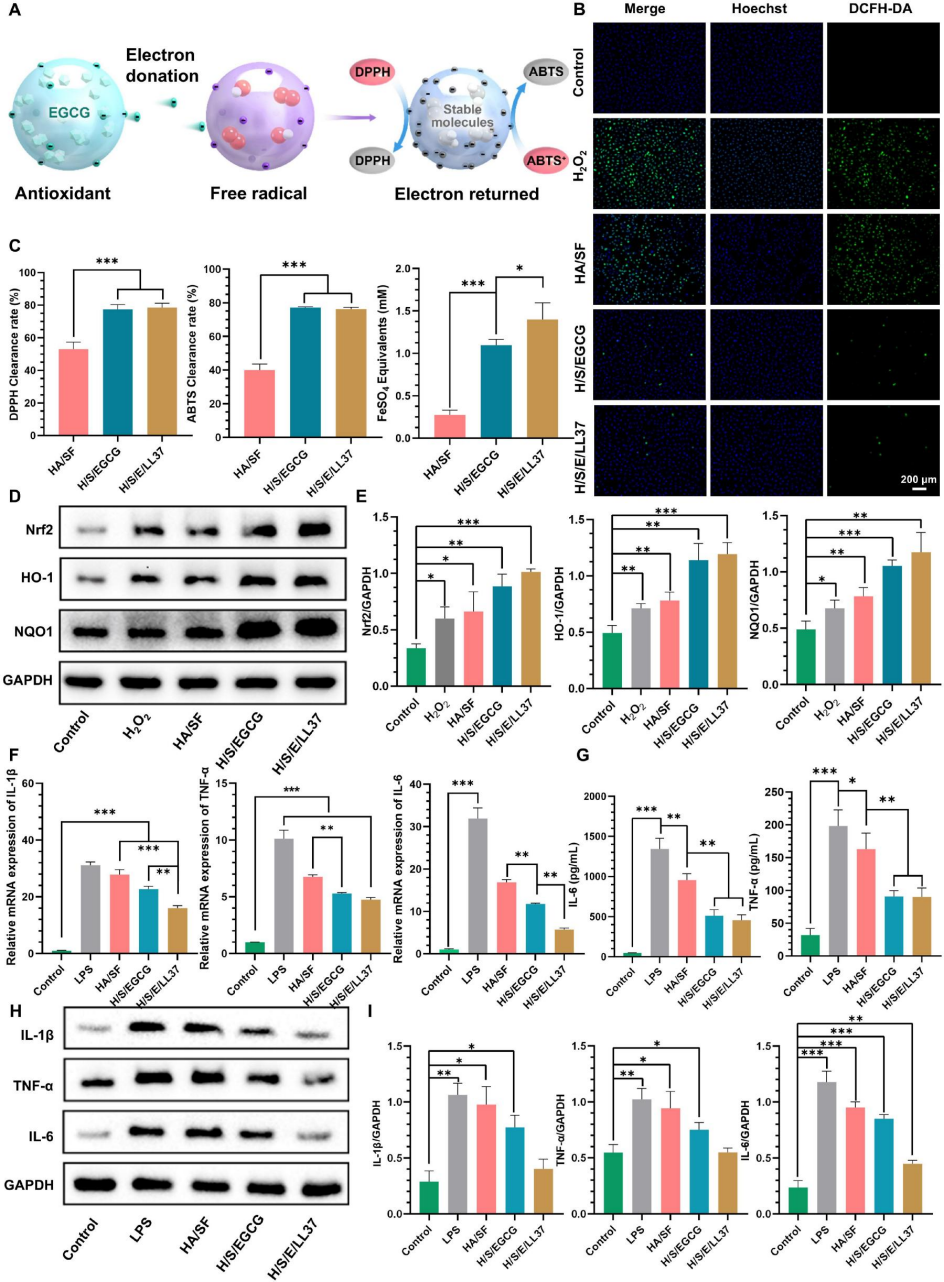
Antioxidant and anti-inflammatory capacities of different hydrogels. (**A**) Schematic diagram illustrating the free-radical scavenging mechanism of EGCG. (**B**) ROS staining (green) in HUVECs treated with LPS and co-cultured with hydrogel scaffold for 24 h; nuclei were counterstained with Hoechst 33342 (blue). Scale bars = 200 μm. (**C**) Evaluation of DPPH radical-scavenging capacity, ABTS^+^ cation-radical-scavenging capacity and total antioxidant capacity (FRAP assay) (*n* = 3). (**D and E**) Western blot analysis of Nrf2, HO-1 and NQO1 protein expression (*n* = 3). (**F**) qRT-PCR analysis of IL-1β, TNF-α and IL-6 mRNA levels (*n* = 3). (**G**) ELISA determination of TNF-α and IL-6 levels (*n* = 3). (**H and I**) Western blot analysis of IL-1β, TNF-α and IL-6 protein expression (*n* = 3). *P < 0.05, **P < 0.01, ***P < 0.001.

When materials are implanted into gingival defect sites, they can easily lead to an increase in ROS. Excessive ROS production causes oxidative stress, which in turn damages biomolecules such as DNA, lipids and proteins, interferes with cell proliferation post-implantation, and hinders tissue regeneration [46]. Delivering antioxidants via tissue engineering scaffolds may help suppress ROS generation, modulate immune responses, and promote tissue regeneration. Studies have confirmed that the phenolic hydroxyl groups of EGCG can provide hydrogen atoms and undergo oxidation reactions to form highly stable compounds, thereby scavenging a large amount of harmful ROS *in vivo* [50] (Figure 5A). We co-cultured scaffolds from each group with HUVECs and L929 cells induced into an oxidative stress state by H_2_O_2_. Intracellular ROS levels in cells from each group were detected using the DCFH-DA probe (Figure 5B and Supplementary Figure S6). Fluorescence microscopy imaging showed that after H_2_O_2_ induction, ROS levels were significantly elevated in the H_2_O_2_-treated group (positive control). Intervention with HA/SF did not significantly improve intracellular ROS levels, whereas intervention with the H/S/EGCG and H/S/E/LL37 groups notably reduced the fluorescence intensity of intracellular ROS in both the groups, suggesting an association with the release of EGCG from the hydrogel scaffolds. The free-radical scavenging capacity of the materials was assessed using ABTS and DPPH scavenging assays. As shown in Figure 5C, all three hydrogel scaffold groups exhibited certain levels of DPPH and ABTS^+^ radical-scavenging rates. The DPPH scavenging rate of the HA/SF hydrogel scaffold alone was only 53.01 ± 4.31%. After incorporating EGCG, the DPPH scavenging rates of the H/S/EGCG and H/S/E/LL37 groups significantly increased to 77.39 ± 2.94% and 78.49 ± 2.70%, respectively. The ABTS^+^ radical-scavenging rates of the three hydrogel scaffold groups showed a similar trend to DPPH, with values of 39.93 ± 3.60%, 77.04 ± 0.63% and 76.24 ± 1.01%, respectively. This demonstrates that the hydrogel scaffolds possess good antioxidant capacity after the addition of EGCG. The results of the FRAP total antioxidant capacity assay also support this conclusion. Additionally, cellular proteins were collected and extracted from each group. Western blot analysis revealed that in both the H/S/EGCG and H/S/E/LL37 groups, the levels of the oxidative stress regulator Nrf2 and its downstream antioxidant proteins HO-1 and NQO1 were significantly elevated, with comparable levels between the two groups (Figure 5D and E). This coordinated upregulation is in line with the well-characterized ability of EGCG to disrupt the Keap1–Nrf2 interaction and activate the Nrf2/ARE antioxidant pathway. Collectively, these results demonstrate that the EGCG-containing scaffolds effectively protect cells from oxidative stress-induced damage through this endogenous defense mechanism [39, 51].

Placing materials in gingival recession areas promotes bacterial infection at the wound site, driving macrophage polarization toward the pro-inflammatory M1 state and upregulating TNF-α, IL-6 and IL-1β. Therefore, to evaluate the ability of the hydrogel scaffolds to modulate inflammatory responses *in vitro*, scaffolds from each group were co-cultured with cells in an inflammatory state induced by LPS. RT-qPCR results showed that LPS-induced RAW 264.7 cells significantly expressed the inflammatory factors IL-1β, TNF-α and IL-6. However, after intervention with H/S/EGCG and H/S/E/LL37, the expression levels of these inflammatory factors were partially reversed. Notably, the improvement in inflammatory and matrix protein levels was more pronounced with H/S/E/LL37 compared to H/S/EGCG (Figure 5F). To further validate the anti-inflammatory effects, we also used ELISA to measure the release of IL-6 and TNF-α in the cell supernatant. It was found that after intervention with H/S/EGCG and H/S/E/LL37, the release of IL-6 and TNF-α in the supernatant was significantly reduced (Figure 5G). Meanwhile, Western blot results yielded conclusions similar to those mentioned above (Figure 5H and I). These results collectively indicate that EGCG and LL-37 can act synergistically to reduce cellular inflammation levels. Furthermore, dynamically regulating the antimicrobial and antioxidant microenvironment is critical for successful periodontal repair [52]. Consistent with this, our supplemented immunofluorescence data (Supplementary Figure S7) explicitly confirm that the H/S/E/LL37 scaffold drives macrophage polarization from a pro-inflammatory M1 (iNOS-positive) to a pro-healing M2 (Arg-1-positive) phenotype. This M2-dominated microenvironment synergistically enhances downstream fibroblast migration and angiogenesis, ultimately accelerating the tissue regeneration observed *in vivo*.

### The HA/SF/EGCG/LL37 hydrogel scaffold demonstrated enhanced healing efficacy in both dorsal skin defect and gingival defect models

**Figure 6:**
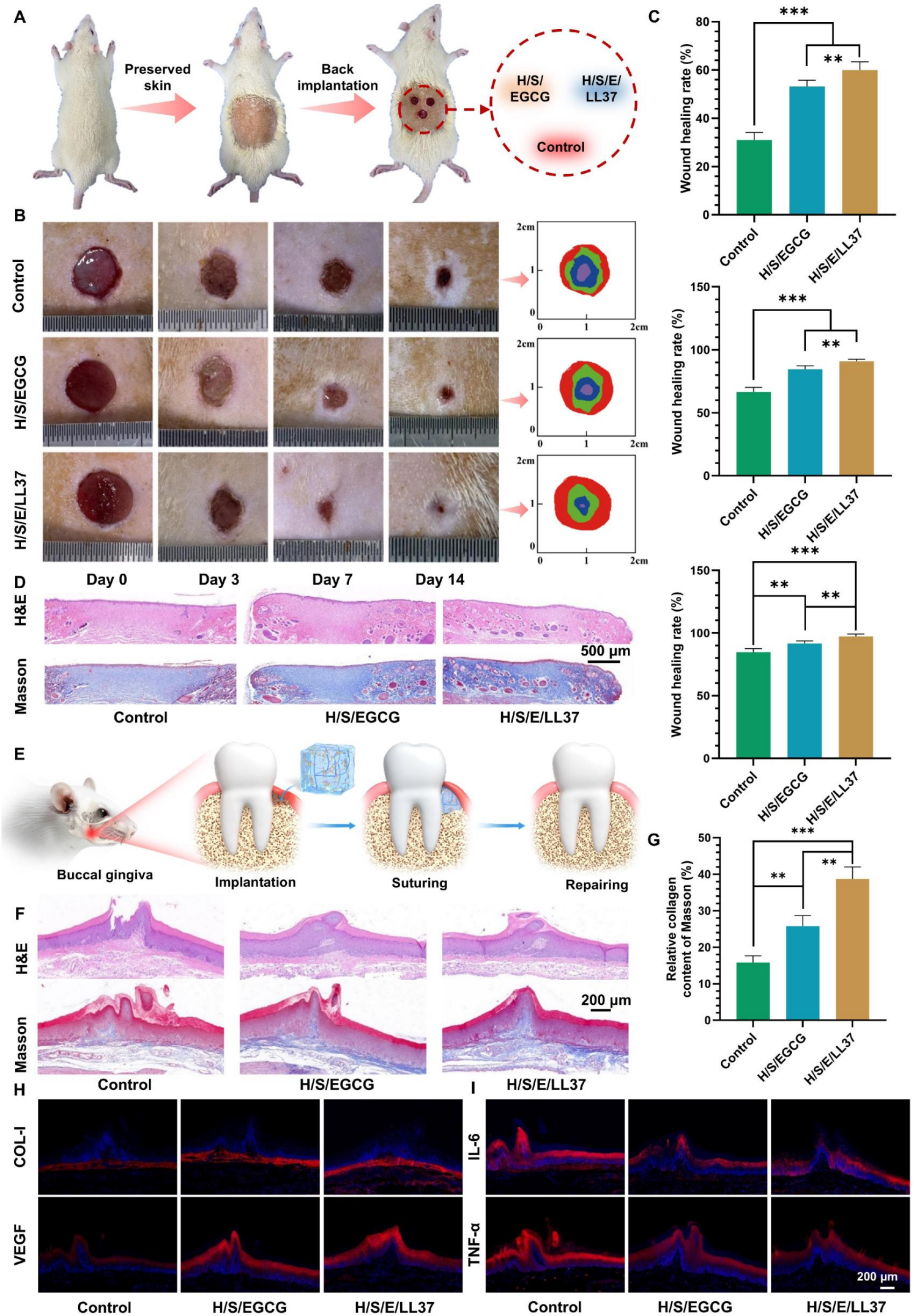
Hydrogel scaffolds promote skin wound healing and gingival defect repair. (**A**) Schematic diagram illustrating the implantation of hydrogel scaffold into a rat dorsal skin wound model. (**B**) Photographs and schematic diagrams of wounds treated with control, H/S/EGCG and H/S/E/LL37 at 0, 3, 7 and 14 days. (**C**) Quantitative analysis of wound area closure (*n* = 5). (**D**) H&E and Masson’s trichrome staining of skin wound sections at day 14. Scale bars = 500 μm. (**E**) Schematic diagram of the rat oral gingival defect model. (**F**) H&E and Masson’s trichrome staining of the rat gingival defect model at day 21. Scale bars = 200 μm. (**G**) Quantitative analysis of collagen deposition at day 21 post-treatment (*n* = 5). (**H**) Immunofluorescence staining for COL-I and VEGF. Scale bars = 200 μm. (**I**) Immunofluorescence staining for IL-6 and TNF-α. Scale bars = 200 μm.*P < 0.05, **P < 0.01, ***P < 0.001.

To evaluate the *in vivo* tissue regeneration potential of the H/S/E/LL37 scaffold, we fabricated it into disk-shaped samples (diameter: 10 mm, thickness: 10 mm) and implanted them into full-thickness skin defects (diameter: 10 mm) created on the dorsal side of male SD rats. The experiment included a PBS control group and an H/S/EGCG scaffold group without LL-37 for comparison (Figure 6A). Over time, wound areas gradually decreased in all groups, with the H/S/E/LL37 group consistently showing the smallest wound areas at days 3, 7 and 14 (Figure 6B). Quantitative analysis revealed that on postoperative day 3, the wound closure rates for the H/S/EGCG and H/S/E/LL37 groups were 24.0 ± 2.3% and 30.7 ± 1.1%, respectively, both significantly higher than that of the control group (14.3 ± 2.9%). Notably, on postoperative day 14, the H/S/E/LL37 group achieved a wound closure rate of 92.3 ± 2.4%, which was markedly superior to the control group (74.9 ± 3.2%) and the H/S/EGCG group (83.9 ± 1.0%) (Figure 6C). Histological analysis by hematoxylin and eosin (H&E) staining further confirmed this trend (Figure 6D). The H/S/E/LL37 group exhibited denser granulation tissue, more pronounced epidermal and dermal regeneration, and the highest degree of wound closure.

Collagen deposition and remodeling are crucial for promoting tissue healing. Masson’s trichrome staining results demonstrated that the H/S/E/LL37 group exhibited more abundant newly formed collagen deposition (Figure 6D). These data collectively suggest that this scaffold holds promising potential for promoting skin defect repair.

Building on these findings, we further evaluated the scaffold’s effect on soft tissue regeneration in a rat gingival defect model. PBS (control), H/S/EGCG scaffolds and H/S/E/LL37 scaffolds were respectively implanted into the surgical wounds (Figure 6E). Analysis of samples harvested 21 days post-implantation showed, as illustrated in Figure 6F, that H&E staining revealed more organized collagen fiber arrangement and a thicker mucosal layer in the H/S/E/LL37 group. Masson’s staining similarly indicated significantly higher collagen expression levels in this group compared to the other two (Figure 6G).

Given that COL‑I is a major component of gingival tissue, we performed immunofluorescence staining for its detection and found stronger positive signals for COL-I in the H/S/E/LL37 group. Neovascularization is also a key aspect of healing, as it ensures nutrient and cellular supply. Therefore, we simultaneously examined VEGF, an important marker for neovascularization. According to immunofluorescence staining results, the H/S/E/LL37 group exhibited higher VEGF expression levels, suggesting a greater pro-angiogenic capacity (Figure 6H). Furthermore, we assessed the inflammatory response during the healing process. Immunofluorescence detection for TNF-α and IL-6 revealed markedly weaker positive signals in the H/S/E/LL37 group compared to the control and H/S/EGCG groups (Figure 6I), indicating that this scaffold also possesses certain anti-inflammatory effects.

Autogenous connective tissue graft remains the clinical gold standard for soft tissue augmentation-proven to increase gingival width and thickness, improve aesthetics and ensure long-term stability [53]. However, it requires a second surgical site, raising risks of infection, pain and swelling [54]. Consequently, researchers are pursuing biomaterial alternatives with strong mechanical properties, biocompatibility, cell adhesion and low immunogenicity. Current options-like collagen membranes-lack sufficient strength, collapse in moist environments, may trigger immune responses and degrade unpredictably [11, 55]. Designing a scaffold that combines optimal biological function with robust structural integrity is thus a key unmet challenge in oral regenerative medicine.

This study addresses key challenges in gingival soft tissue regeneration-achieving efficient antibacterial activity, precise immunomodulation and functional tissue regeneration within the complex gingival microbiome. To this end, we developed and evaluated a HA/SF hydrogel co-loaded with EGCG and antimicrobial peptide LL-37. The scaffold provides structural support, controlled release, broad-spectrum antibacterial action, pro-angiogenic effects and anti-inflammatory/antioxidant activity-collectively establishing a dynamically regulatable microenvironment that promotes gingival soft tissue regeneration. The scaffold was built on an EDC/NHS-cross-linked porous HA/SF base, offering suitable mechanical strength, controllable swelling and a porous structure that supports cell migration and nutrient exchange. EGCG and LL-37 were loaded and functionally validated to confer bioactivity. Unlike typical burst release from physical adsorption, LL-37 showed sustained, controllable release-likely due to electrostatic attraction between its positive charge and EGCG’s negative charge-maintaining therapeutic concentrations at the wound site during critical healing stages. The scaffold also exhibited potent, broad-spectrum antibacterial activity against *S. aureus*, *E. coli*, and *P. gingivalis* (a key periodontal pathogen), as confirmed by live/dead staining, CFU counts, SEM and biofilm inhibition assays. Its synergy arises from LL-37 disrupting bacterial membranes and EGCG interfering with membrane potential and key enzyme activity-crucial for preventing secondary infections in microbially complex gingival recession sites. The scaffold exhibits broad-spectrum antibacterial activity, excellent biocompatibility and robust pro-regenerative functions. The superior regenerative performance of the H/S/E/LL37 scaffold is attributed to a sophisticated synergistic crosstalk between EGCG and LL-37 at the signaling level. EGCG primarily functions to ‘reset’ the hostile microenvironment by activating the Nrf2/HO-1 antioxidant pathway [51], which effectively scavenges excessive ROS and resolves local oxidative stress. This cleared microenvironment provides a permissive state that allows LL-37 to more efficiently engage its receptors (FPR2 and EGFR) and activate downstream MAPK/ERK and PI3K/Akt signaling pathways [35]. In the absence of EGCG-mediated antioxidant protection, these pro-regenerative signals are often dampened by oxidative damage. Together, EGCG and LL-37 synergistically drive the rapid polarization of macrophages from a pro-inflammatory M1 phenotype to a reparative M2 phenotype, which is a critical switch for transitioning the wound from acute inflammation to functional tissue remodeling and angiogenesis*. In vivo*, the H/S/E/LL37 scaffold accelerated wound closure, granulation tissue formation and collagen deposition in a rat skin wound model. In a rat gingival defect model-a more clinically relevant setting-it promoted regeneration of thicker, well-organized gingival tissue, with increased COL‑I and VEGF expression, confirming dual enhancement of soft-tissue repair and angiogenesis. Furthermore, it is important to note that true periodontal restoration requires more than just isolated soft tissue healing; it necessitates the functional reconstruction of the complex periodontal attachment apparatus. As highlighted in recent literature, precise regulatory mechanisms and targeted regeneration of the soft–hard tissue interface represent the goal for periodontium repair [56]. While our current study primarily focuses on modulating the soft tissue microenvironment and promoting volumetric healing through a rat gingival defect model, this immunomodulatory and biologically active scaffold lays a necessary foundation for future advanced designs targeting the integrated soft-hard tissue interface in true root-exposure animal models. Concurrently, local TNF-α and IL-6 levels were significantly reduced, consistent with its *in vitro* anti-inflammatory effects.

This study shows that the H/S/E/LL37 hydrogel scaffold offers a promising translational solution for gingival soft tissue regeneration. Beyond acting as a physical barrier and antibacterial agent, it actively guides fibroblast migration and attachment, promotes angiogenesis and shifts macrophages toward a pro-reparative phenotype-systematically supporting soft tissue regeneration and tissue homeostasis.

## Conclusion

In this study, we successfully designed and characterized a multifunctional H/S/E/LL37 hydrogel scaffold for oral soft tissue repair. The scaffold exhibited a unique porous microstructure, controllable degradability, sustained release of LL-37 and favorable biocompatibility. *In vitro*, it demonstrated broad-spectrum antibacterial activity, potent antioxidant and anti-inflammatory effects, and pro-angiogenic capacity. *In vivo*, implantation of the scaffold in rat skin and gingival defect models significantly promoted angiogenesis, collagen deposition and wound closure while reducing local inflammation.

Importantly, the H/S/E/LL37 scaffold holds considerable translational potential as an ‘off-the-shelf’ candidate that could simplify surgical workflows by eliminating the need for a palatal donor-site, thereby mitigating donor-site morbidity. However, it should be noted that the current *in vivo* evaluation did not employ a true gingival recession/root-exposure model; therefore, further validation in clinically representative models that recapitulate root exposure and attachment loss will be necessary to fully establish its efficacy for periodontal soft tissue regeneration. In summary, this multifunctional scaffold represents a promising biomaterial platform that warrants future investigation for oral soft tissue augmentation and repair.

## Supplementary Material

rbag131_Supplementary_Data

## Data Availability

The data that support the findings of this study are available from the corresponding author upon reasonable request.
