# Supplementary material for: A degradable hydrogel scaffold incorporating the LL-37 for gingival soft tissue regeneration
Source: Regen Biomater. 2026 Jun 25;13:rbag131. doi: 10.1093/rb/rbag131 (PMC13363250; doi:10.1093/rb/rbag131)
Supplement: rbag131_Supplementary_Data [file rbag131_supplementary_data.docx]

**Supplementary Information**

**A Degradable Hydrogel Scaffold Incorporating the Antimicrobial Peptide LL-37 for Gingival Soft Tissue Augmentation**

Jing Mao et al.

**Supplementary figures and figure legends**

**S1**

**Figure S1.** G′ and G″ vs. angular frequency (0.1–100 rad/s) for fully swollen scaffolds at 37 °C.

**S2**


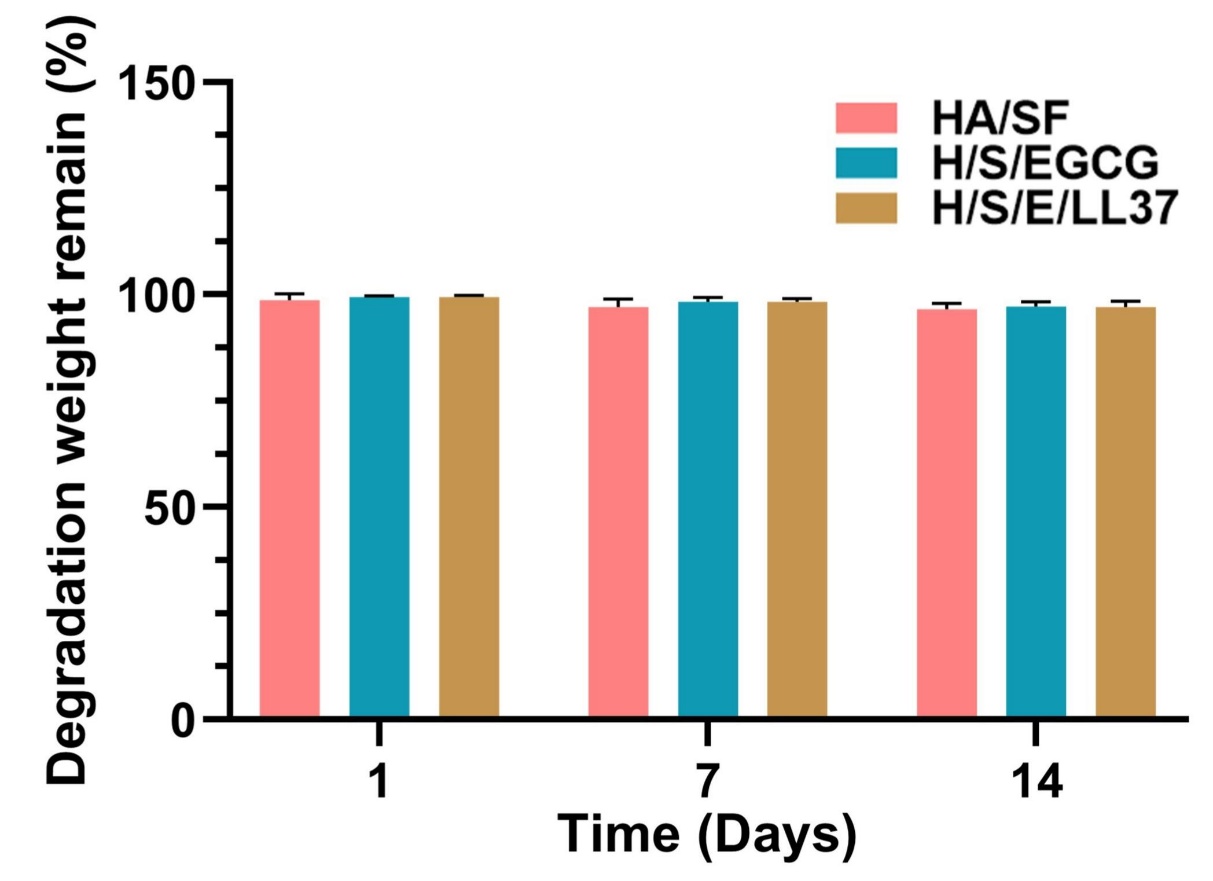


**Figure S2.** Degradation loss of hydrogel scaffolds in PBS solution at different time points (n = 3).

**S3**


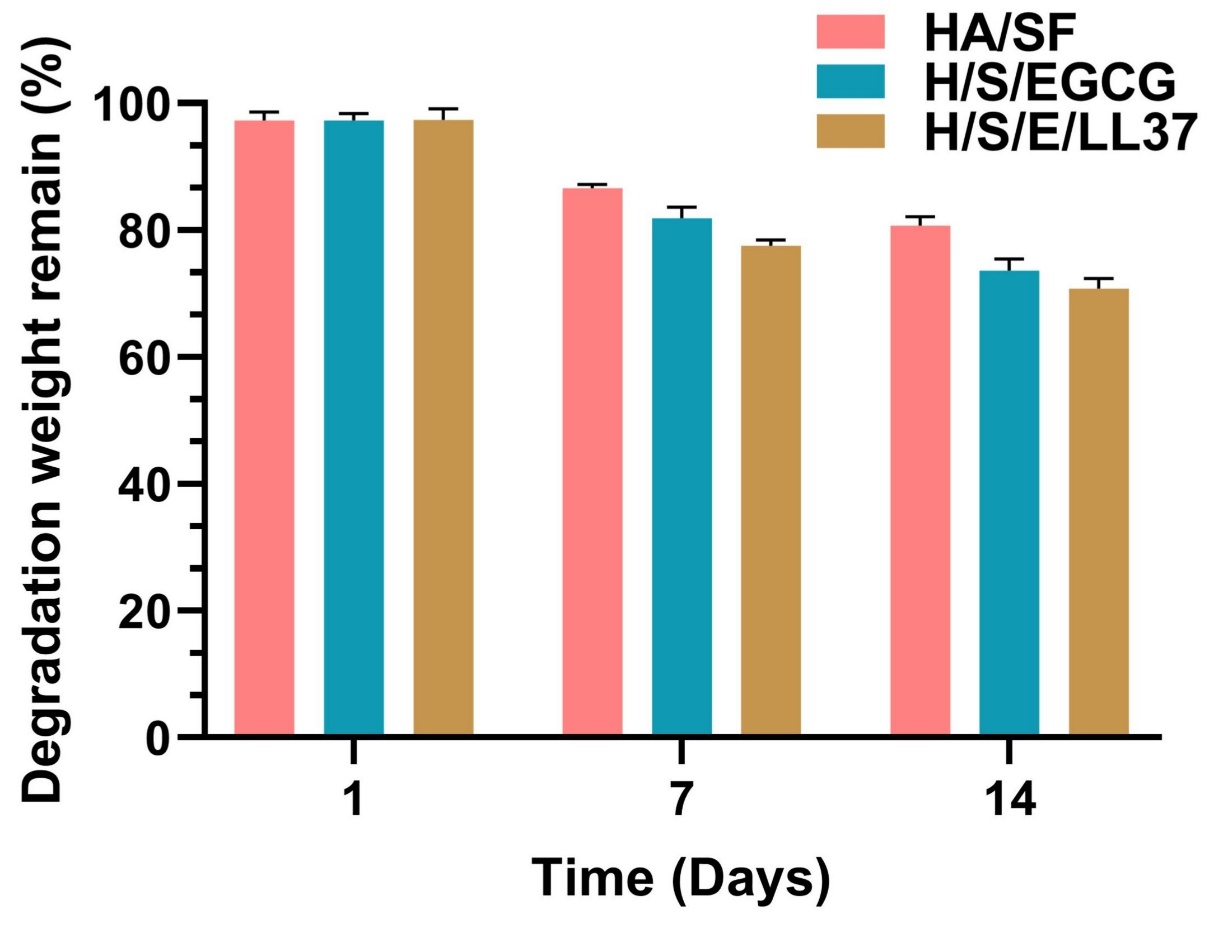


**Figure S3.** Mass retention of scaffolds under dynamic shaking in artificial saliva (pH 6.8, 60 rpm, 37 °C) at 1, 7, and 14 days, with periodic medium change. Mean ± SD (n = 3).

**S4**


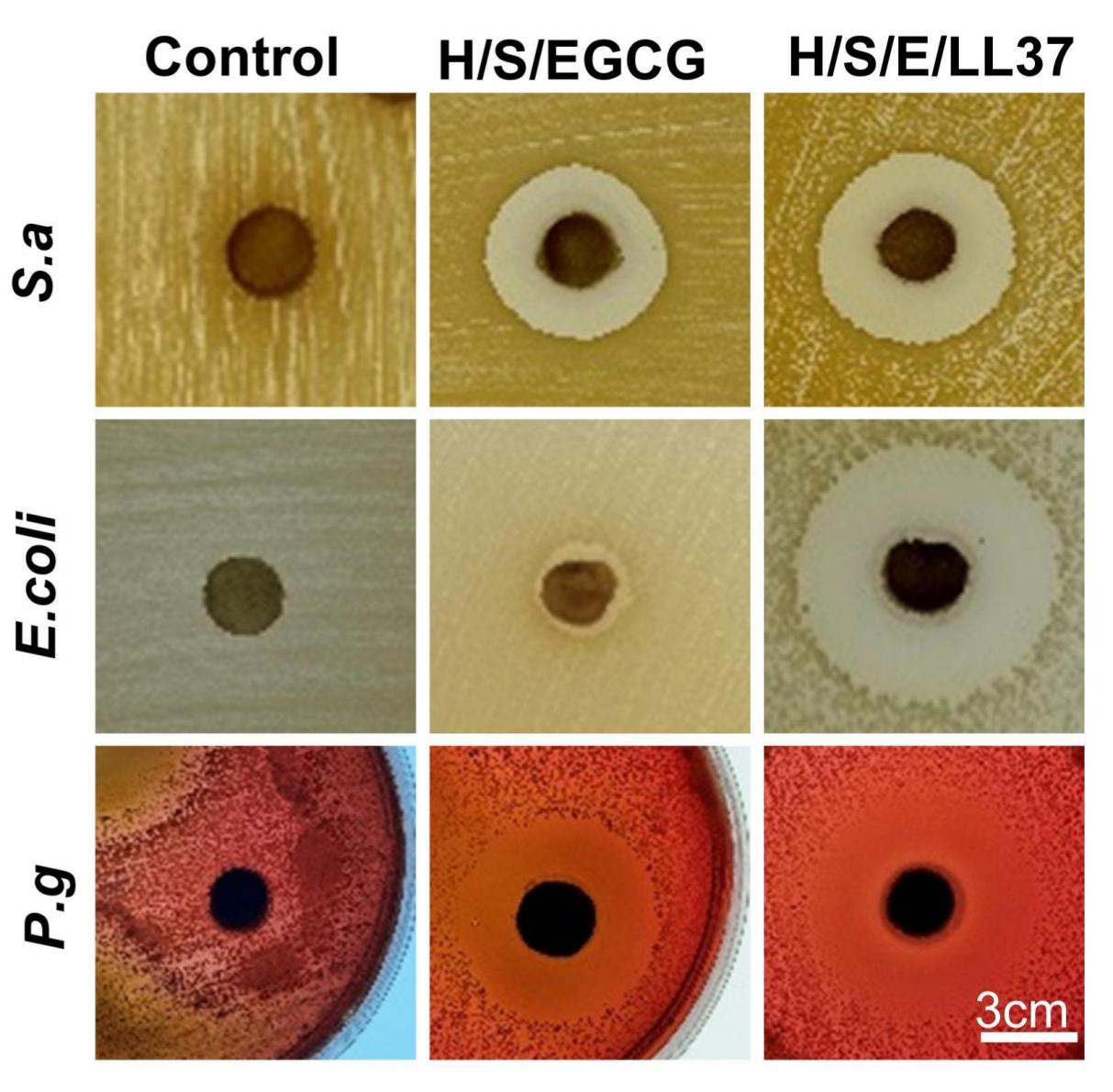


**Figure S4.** Zone of inhibition before and after sample release. Scale bar =3 cm.

**S5**


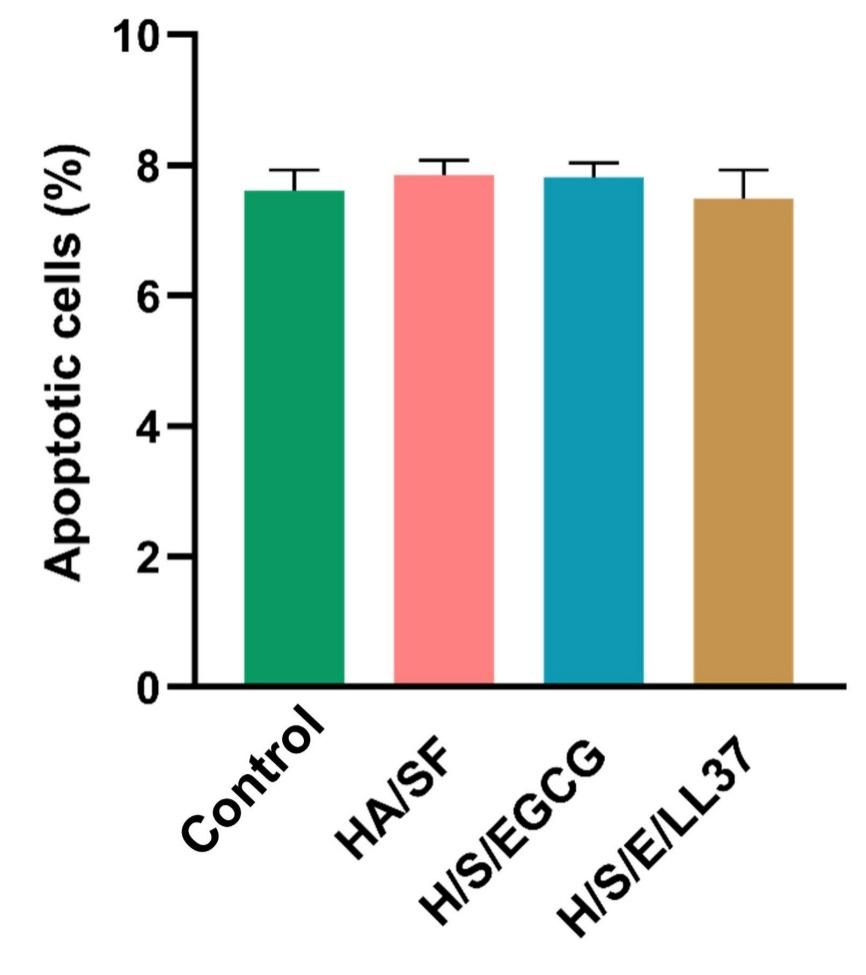


**Figure S5.** Analysis of scaffold-induced apoptosis in L929 cells.

**S6**


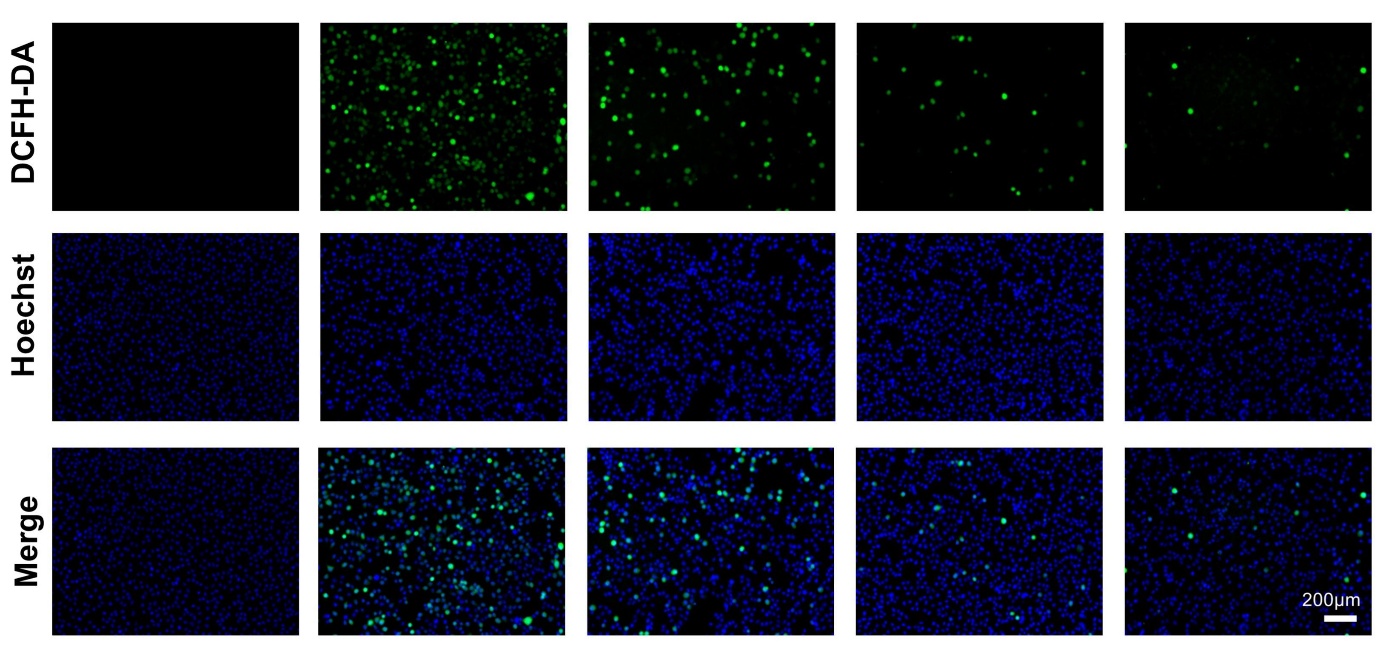


**Figure S6.** Intracellular ROS staining (green) in L929 cells treated with H₂O₂ and co-cultured with hydrogel materials for 24 h; nuclei were counterstained with Hoechst 33342 (blue). Scale bar = 200 μm.

**S7**


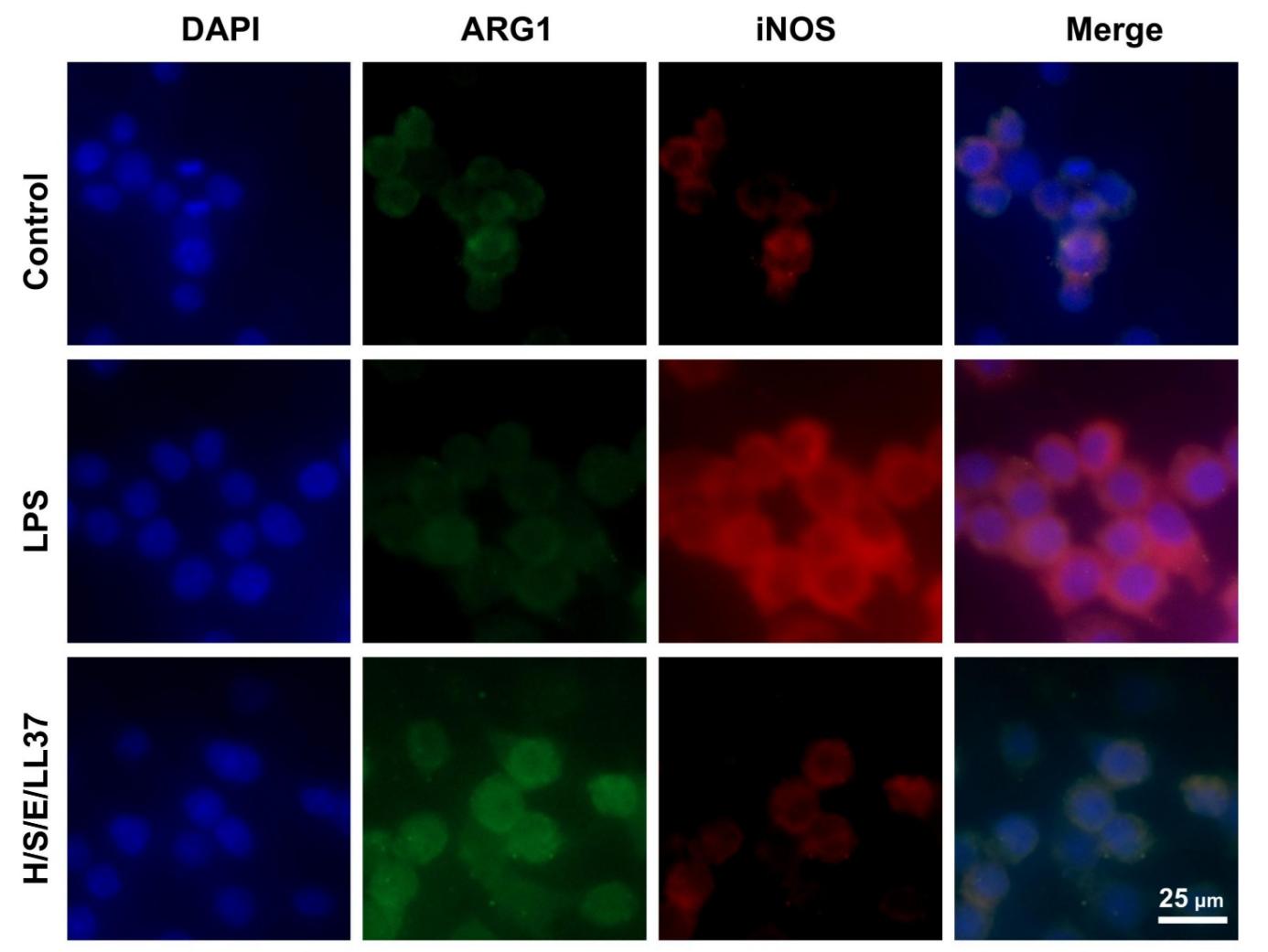


**Figure S7.** Immunofluorescence staining of Arg‑1 (M2 marker, green) and iNOS (M1 marker, red) in RAW 264.7 macrophages under Control, LPS treatment, and LPS + H/S/E/LL37 scaffold co‑culture. Scale bar = 25 μm.
